# Author Correction: Ivermectin converts cold tumors hot and synergizes with immune checkpoint blockade for treatment of breast cancer

**DOI:** 10.1038/s41523-026-00908-1

**Published:** 2026-02-27

**Authors:** Dobrin Draganov, Zhen Han, Aamir Rana, Nitasha Bennett, Darrell J. Irvine, Peter P. Lee

**Affiliations:** 1 Department of Immuno-Oncology, Beckman Research Institute, City of Hope, Duarte, CA USA; 2 Koch Institute for Integrative Cancer Research and Department of Biological Engineering, Massachusetts Institute of Technology, Cambridge, MA USA; 3 Howard Hughes Medical Institute, Chevy Chase, MD USA

Correction to: *npj Breast Cancer* 10.1038/s41523-021-00229-5, published online 02 March 2021

In this article, errors were discovered in Figure 4 panels D and F. Because the original data are no longer available to replace the incorrect images and it isn’t feasible to repeat the experiments, the incorrect images have been blocked out. The overall results and conclusions have not changed. The original article has been corrected.

