# Editorial Expression of Concern: Ivermectin converts cold tumors hot and synergizes with immune checkpoint blockade for treatment of breast cancer

**DOI:** 10.1038/s41523-026-00988-z

**Published:** 2026-06-05

**Keywords:** Breast cancer, Cancer immunotherapy

Editorial Expression of Concern to: *npj Breast Cancer* 10.1038/s41523-021-00229-5, published online 02 March 2021

The Editor would like to alert the readers that concerns have been raised regarding multiple highly similar images between Fig. 4d and f. The authors issued a Correction to exclude some of the duplicate data^1^; however, further checks by the Publisher found further similarities (Fig. 4d IVM mouse 3 and f IVM+aPD1 mouse 1; Fig. 4d aPD1 mouse 2 and 4f IVM+aPD1 mouse 5; Fig. 4d aPD1 mouse 4 and f IVM mouse 5).

The authors have stated that the original data are no longer available. Readers are therefore advised to interpret these data with caution.

Dobrin Draganov, Aamir Rana, Nitasha Bennett, Darrell J. Irvine and Peter P. Lee agree to this Editorial Expression of Concern. The publisher has not been able to obtain a current email address for Zhen Han.

## References

[1] Draganov, D. et al. Author Correction: Ivermectin converts cold tumors hot and synergizes with immune checkpoint blockade for treatment of breast cancer. *npj Breast Cancer* **12**, 31. 10.1038/s41523-026-00908-1 (2026). 41760676 10.1038/s41523-026-00908-1 PMC12949128

