# Supplementary material for: Ivermectin converts cold tumors hot and synergizes with immune checkpoint blockade for treatment of breast cancer
Source: NPJ Breast Cancer. 2021 Mar 2;7:22. doi: 10.1038/s41523-021-00229-5 (PMC7925581; doi:10.1038/s41523-021-00229-5)
Supplement: Supplementary file 1 — Supplementary Materials [file 41523_2021_229_MOESM1_ESM.pdf]

Supplementary Materials

Supplementary Table 1. Experimental Design, Treatment Settings

	Surgical Resection of Tumor	Ivermectin Alone	Anti-PD1 Antibody Alone	Combined Treatment	No Treatment	Days of Observation
Primary	No					
Experiment A		5	0	0	5	25
Experiment B		5	0	0	5	21
Experiment C		5	5	5	5	21
Experiment D		0	5	5	5	49
Experiment E		5	0	5	5	56
Total (N=70)		20	10	15	25	
Neoadjuvant						
With IL-2	Yes		5 (Anti-PD1 + MSA-IL-2)	5#	5	84
Adjuvant	Yes					84
With IL-2		0	0	10	0	
Without IL-2		10	10	10	10	
Total (N=50)		10	10	20	10	
Metastatic	Yes					82
With IL-2		5	5	10	0	
Without IL-2		5	5	5	5	
Total (N=40)		10	10	15	5	

The IP+IVM treatment group shown in Fig. 3A had n=5 with 1 death from surgery complication — thus survival was shown for n=4.

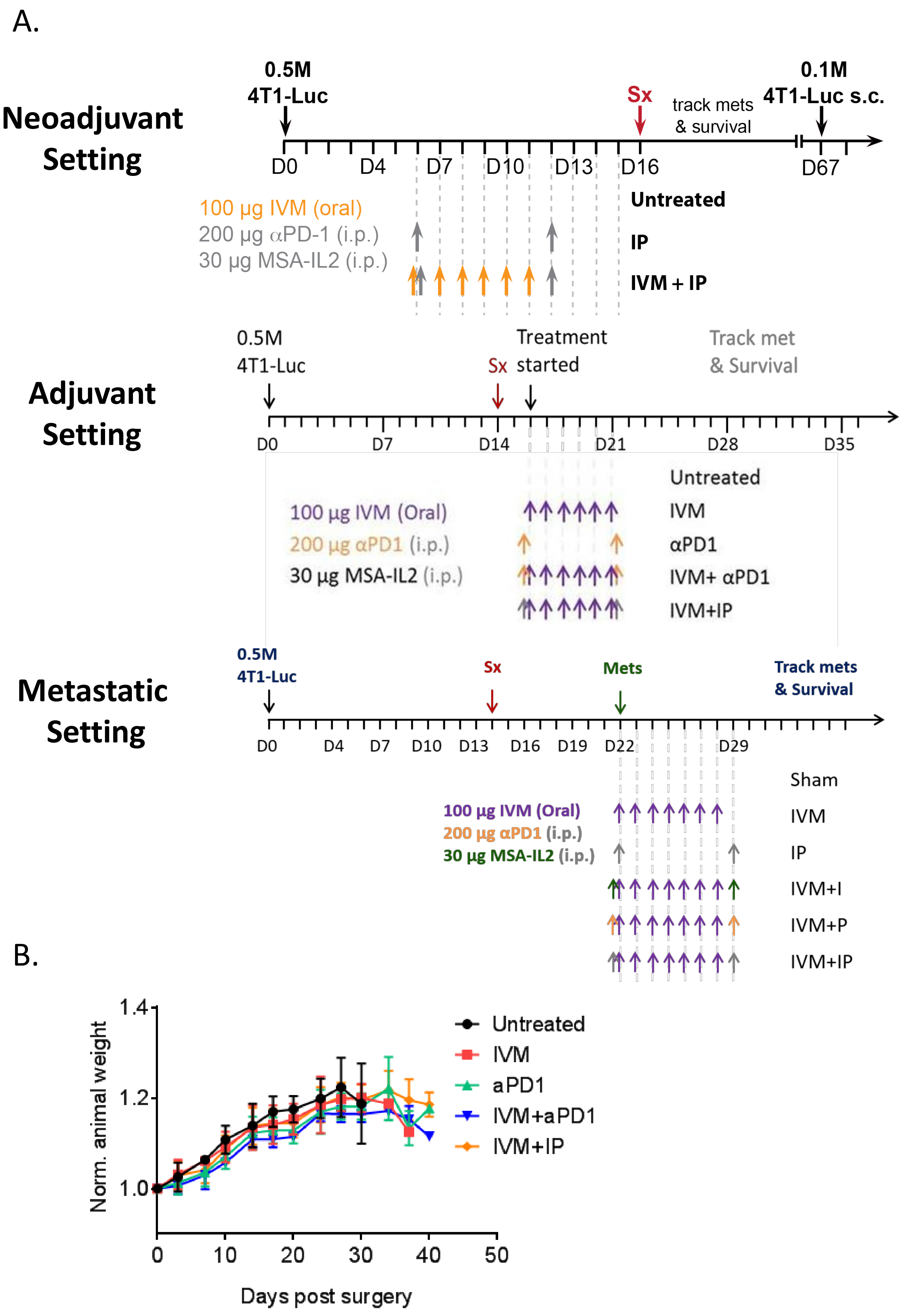
A.
Neoadjuvant Setting
0.5M 4T1-Luc
Sx
track mets & survival
0.1M 4T1-Luc s.c.
D0
D4
D7
D10
D13
D16
D67
100 µg IVM (oral)
200 µg αPD-1 (i.p.)
30 µg MSA-IL2 (i.p.)
Untreated
IP
IVM + IP
Adjuvant Setting
0.5M 4T1-Luc
Sx
Treatment started
Track met & Survival
D0
D7
D14
D21
D28
D35
100 µg IVM (Oral)
200 µg αPD1 (i.p.)
30 µg MSA-IL2 (i.p.)
Untreated
IVM
αPD1
IVM+ αPD1
IVM+IP
Metastatic Setting
0.5M 4T1-Luc
Sx
Mets
Track mets & Survival
D0
D4
D7
D10
D13
D16
D19
D22
D29
100 µg IVM (Oral)
200 µg αPD1 (i.p.)
30 µg MSA-IL2 (i.p.)
Sham
IVM
IP
IVM+I
IVM+P
IVM+IP
B.
Norm. animal weight
1.0
1.2
1.4
0
10
20
30
40
50
Days post surgery
Untreated
IVM
aPD1
IVM+aPD1
IVM+IP

SF1

Supplementary Figure 1. (A) Treatment schedules in the neoadjuvant, adjuvant, and metastatic settings (surgical resection = **Sx**). (B) Body weight measurements of treated animals demonstrating the absence of significant synergistic toxicity associated with the combination of anti-PD-1 and Ivermectin in the adjuvant settings. Similar observations were seen in the metastatic treatment settings.

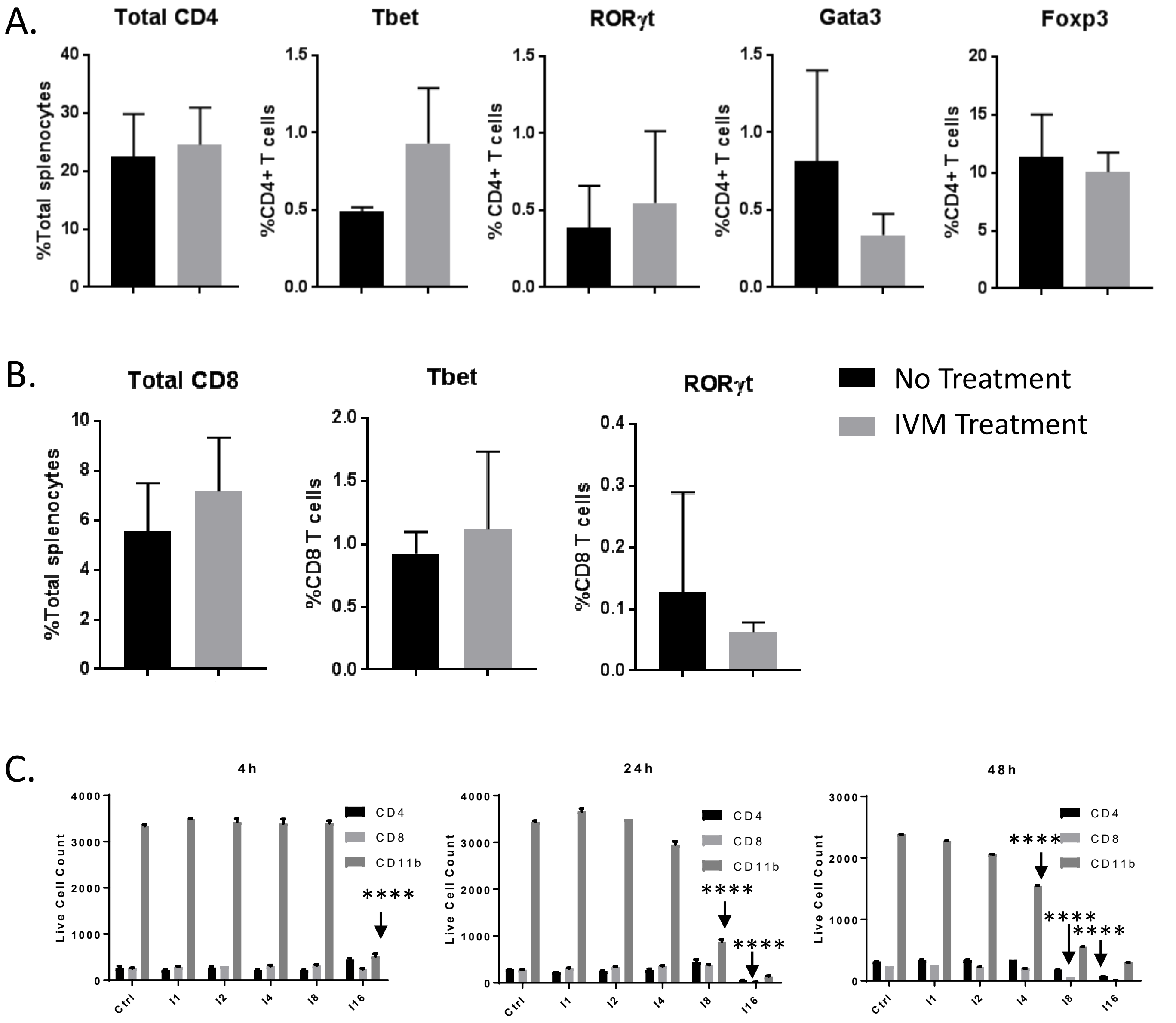
A.
Total CD4
%Total splenocytes
Tbet
%CD4+ T cells
RORγt
% CD4+ T cells
Gata3
%CD4+ T cells
Foxp3
%CD4+ T cells
B.
Total CD8
%Total splenocytes
Tbet
%CD8 T cells
RORγt
%CD8 T cells
No Treatment
IVM Treatment
C.
4h
24h
48h
Live Cell Count
CD4
CD8
CD11b
****
Ctrl
I1
I2
I4
I8
I16

Supplementary Figure 2. Immunomodulatory effects of Ivermectin on immune cells *in vivo* and *ex vivo*. (A) Flow cytometry analysis of splenocytes from 4T1 tumor-bearing animals treated with ivermectin, demonstrating the absence of significant changes *in vivo* of various CD4 (A) and CD8 (B) effector and regulatory T cell subpopulations, which were identified based on the expression of key transcriptional factors as indicated. The transcriptional factors used are well accepted as the most specific markers for different CD4 and CD8 T cell subsets: Tregs (Foxp3+ CD4 T cells), Th1 (Tbet+ CD4 T cells) / Th2 (Gata3+ CD4 T cells) / Th17 (RORgt+ CD4 T cells) / Tc1(Tbet+ CD8 T cells) / Tc17 (RORgt+ CD8 T cells). All comparisons were non-significant, NS. (C) Differential sensitivity of immune subpopulations in splenocytes isolated from 4T1 tumor-bearing mice exposed *ex vivo* to increasing (1-16 μM) doses of ivermectin for 4h to 48h showing dose and time-dependent sensitivity. A linear mixed effects model of log cell count adjusted for cell type revealed that the CD11b+ myeloid cells were the most sensitive to ivermectin, showing significant reductions with as little as 4 μM after 48 hours, or 8 μM after 24 hours, or 16 μM after 4 hours (each result, $p<0.0001$). In contrast, achieving similar reductions in lymphocytes required higher doses and/or longer exposure to ivermectin, being observed in CD8+ cells only after 48 hours of 8 μM or 24 hours of 16 μM and in CD4+ cells only after the maximum exposure (48 hours of 16 μM). Statistical significance versus (-) CTRL or as indicated was evaluated using the linear mixed effects model of log cell count adjusted for cell type, **** $p \leq 0.0001$.

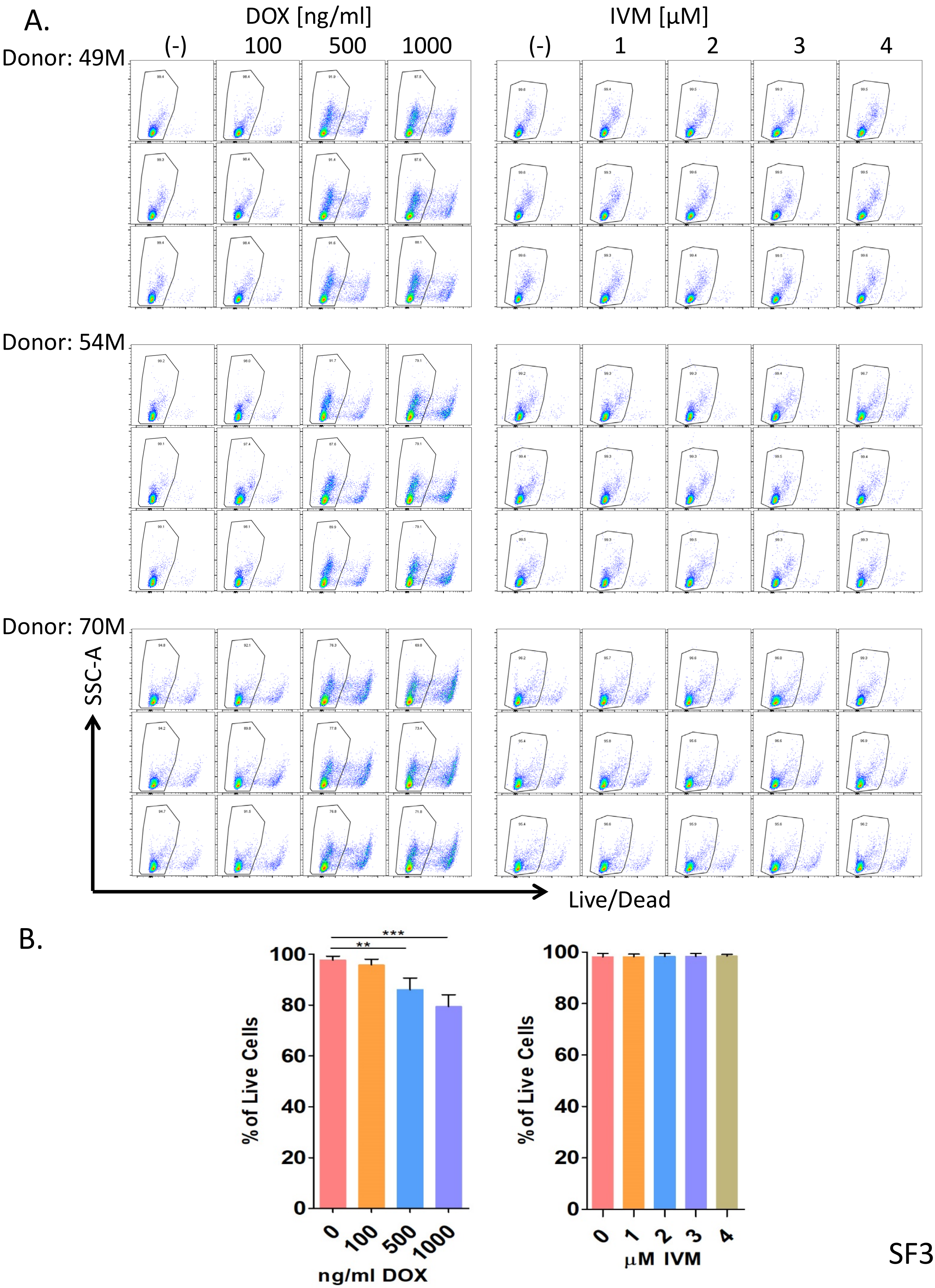
A.
DOX [ng/ml]
(-)
100
500
1000
IVM [μM]
(-)
1
2
3
4
Donor: 49M
Donor: 54M
Donor: 70M
SSC-A
Live/Dead
B.
***
**
% of Live Cells
0
20
40
60
80
100
0
100
500
1000
ng/ml DOX
% of Live Cells
0
1
2
3
4
μM IVM

SF3

Supplementary Figure 3. Cytotoxic effects of physiologically relevant levels of doxorubicin (Dox) and ivermectin (IVM) against human PBMC *ex vivo*. (A) Flow cytometry analysis of the cytotoxic effects of physiologically relevant doses of doxorubicin and Ivermectin, as reported in the literature, against freshly isolated human PBMC treated for 24h *in vitro*. Peripheral blood from three healthy donors was obtained by venipuncture using heparin collection tubes, transported at room temperature from the clinic to the lab and processed within 6 hours of drawing. PBMCs were isolated via Ficoll-Paque gradient centrifugation (GE Healthcare) following the manufacturer's instruction. One million PBMCs in triplicate from each healthy donor were individually treated with 100, 500, or 1000 ng/ml of Doxorubicin (DOX) or 1, 2, 3 or 4 µM of Ivermectin (IVM) in RPMI medium (Life Technologies, Thermo Fisher Scientific) containing 10% FBS for 24h at 37°C with 5% CO2. After 24h of incubation cells were washed once with 1X PBS and stained with Live/DeadTM Fixable Far Red Dead Cell Satin Kit (L34974, Invitrogen, USA,) for 30 minutes in dark at 4°C. Before FACS analysis, cells were washed once with 1X PBS and fixed with 4% PFA. Samples were acquired using a BD Fortessa using FACSDiva 6.1.3. (B) Statistical analysis of the data presented in (A) was performed by using GraphPad Prism version 5 (GraphPad Software Company Incorporation). Values were expressed as mean ± SEM. Statistical significance was determined by using two-way ANOVA followed by Bonferroni post-tests. Statistical differences were considered significant when the P-value was <0.05.
